# Eating Veggies Is Fun! An Implementation Pilot Study in Partnership With a YMCA in South Los Angeles

**DOI:** 10.5888/pcd15.180150

**Published:** 2018-11-01

**Authors:** Annette E. Maxwell, Laura Castillo, Anthony A. Arce, Teresa De Anda, David Martins, William J. McCarthy

**Affiliations:** 1 Center for Cancer Prevention and Control Research, University of California, Los Angeles, Fielding School of Public Health, and Jonsson Comprehensive Cancer Center, Los Angeles, California; 2 Department of Medicine, College of Medicine, Charles R. Drew University, Los Angeles, California; 3 Department of General Internal Medicine, David Geffen School of Medicine at UCLA, Los Angeles, California

## Abstract

**Purpose and Objectives:**

Children eat less than recommended amounts of vegetables. Repeated taste exposure can increase children’s acceptance of initially disliked vegetables. However, implementation of this strategy is lacking. We conducted a pilot study to assess the feasibility of implementing an evidence-based intervention to promote liking of initially disliked vegetables among children enrolled in a YMCA summer camp.

**Intervention Approach:**

We adapted a research-tested intervention to promote child liking of vegetables for implementation in small groups. In summer 2015, 50 children aged 7 to 12 years were invited to taste 5 initially disliked vegetables daily for 10 days.

**Evaluation Methods:**

Children rated how much they liked vegetables on a 5-point emoji-like faces Likert scale at baseline and 2- and 4-week follow-up. The mean ratings for liked and initially disliked vegetables were estimated over time using mixed effects modeling.

**Results:**

We achieved excellent participation of parents and children; however, we experienced nonstudy-related attrition caused by disenrollment of some children from the weekly camp program. The average liking increased over time (linear trend, *P* = .003) for the 5 targeted vegetables but not for the other nontargeted vegetables, as predicted.

**Implications for Public Health:**

This pilot study suggests that repeated vegetable tasting opportunities offered by community programs may be a practical strategy for introducing low-income, young children to new or initially disliked vegetables. The study demonstrates the feasibility of implementing a health promotion strategy that has the potential to improve population health in a community setting in an underresourced neighborhood.

## Purpose and Objectives

Most children eat less than the federally recommended daily amount of vegetables (1). Getting children to eat recommended amounts of minimally processed fruits and vegetables daily appears to be an important influence on their achieving and maintaining a healthy weight (2) and reducing their later-in-life risk of major chronic diseases such as cardiovascular disease, diabetes, various cancers, and stroke (3–5).

Having children eat more minimally processed fruits and vegetables leads to reduced consumption of energy-dense foods in families (6), although this may be more true of children from middle- and high-income families than from low-income families (7). Many children, especially those in low-income families, are not familiar with different types of vegetables and typically report not liking their taste when first exposed to a new type (8,9). The greater the child’s knowledge about different types of vegetables, the greater the child’s vegetable intake (10). Providing a variety of fruits and vegetables can lead to increased consumption of both food types in children (11). Increased consumption of fruits and vegetables in childhood is associated with decreased risk of cancer and stroke in later life (4,5). Randomized controlled trials have demonstrated that repeated taste exposure can increase children’s acceptance of initially disliked vegetables (12–14). However, implementation of this strategy is lacking. We conducted a pilot study to assess the feasibility of implementing an evidence-based intervention to promote liking of initially disliked vegetables among children enrolled in a YMCA summer camp in South Los Angeles.

South Los Angeles is part of Service Planning Area (SPA) 6 in Los Angeles County, where residents are mostly African American (28.5%) or Latino (67.7%). Of the 8 SPAs in Los Angeles County, SPA 6 has the largest proportion of adults with less than a high school education (41.6%) and a household income of less than 100% of the federal poverty guidelines (33.6%). It has the lowest number of adults who consume 5 or more servings of fruits and vegetables a day (9.6%) and the highest proportion of adults who are classified as obese (34.1%) (15).

## Intervention Approach

To promote healthier eating, we adapted a research protocol from a trial originally conducted in British kindergartens and elementary schools (16) called Tiny Tastes. A description of Tiny Tastes was subsequently posted on the National Cancer Institute’s Research-Tested Intervention Programs (RTIPs) website as a research-tested intervention program recommended for dissemination (17). RTIPs is a searchable database of evidence-based cancer control interventions and program materials and is designed to provide program planners and public health practitioners easy and immediate access to research-tested materials. RTIPs-listed programs are effective in the populations and settings in which they were studied. Many researchers are advocating for the adoption, adaptation, and implementation of these programs because their documented past success makes it more likely that they will yield cancer control benefit in new populations than a newly created program that has not been tested anywhere (18).

The Tiny Tastes program repeatedly exposes children to small bites of initially disliked vegetables to increase their liking for these vegetables. Our pilot study objectives were to 1) assess the feasibility of adapting the evidence-based intervention and implementing it in the busy environment of a YMCA summer camp, and 2) explore the short-term impact of the program on children’s liking of initially disliked vegetables and on their willingness to try new foods.

## Evaluation Methods

### Partnership with a local YMCA site

In North America, the YMCA is primarily a community sports facility, open to all, regardless of religion, social class, sex, or age (19). The 2,700 separate YMCA entities in North America focus on “nurturing the potential of every child and teen, improving the nation’s health and well-being and providing opportunities to give back and support neighbors” (19). The Weingart YMCA Wellness and Aquatic Center in South Los Angeles agreed to host the proposed study, recognizing that the goal to increase local children’s vegetable intake was consistent with its mission. This site was already partnering with a local university, dedicating classroom space and a test kitchen within its facility to nutrition research. The intervention and all assessments took place in this classroom space.

### Recruitment and informed consent

Participants were recruited from the Weingart YMCA Wellness and Aquatic Center in South Los Angeles from a pool of 109 children aged 7 to 12 years who were enrolled in the summer day camp. YMCA summer day camps provide a safe and enriching environment for children to participate in various healthy, developmentally appropriate activities and learning experiences when school is not in session. Generally, campers are divided into smaller groups to participate in structured activities, including sports, and receive morning and afternoon snacks and lunch.

Bilingual research staff passed out information about the study in English and Spanish to parents who dropped off their children in the morning, including a form that parents could mark to opt their child(ren) out of the study (passive consent). All children spoke English. At scheduled times, the camp director brought small groups of age-eligible, study-eligible children to the test kitchen where research staff explained the study and asked if children wanted to participate. The study was approved by the University of California, Los Angeles (UCLA), Institutional Review Board.

### The original research-tested intervention: Tiny Tastes

Tiny Tastes is an effectiveness trial posted on RTIPS that showed in a randomized, controlled design that multiple exposures to initially disliked vegetables increased preschool children’s liking for those vegetables in the intervention group more than in a no-treatment control group (*P* < .001). The Tiny Tastes program (16,17) had each child (aged 4–6 years) rate how much they liked 6 vegetables (carrot, red bell pepper, sugar snap pea, cabbage, cucumber, and celery) using a 3-point scale with facial expressions corresponding to yummy, just okay, and yucky. For each child, the vegetable that they had ranked number 4 (moderately disliked) became their target vegetable. Each child tasted a small piece of their target vegetable in 12 individual sessions over 3 weeks. In this randomized controlled trial, 1 group of children received tangible rewards (stickers) for tasting the target vegetable, a second group received social rewards (praise for tasting) and a third group received neither. Each child rated liking of their target vegetable on day 15 and at 1- and 3-month follow-up visits. On day 15, all 3 groups achieved a greater increase in liking of the target vegetable compared with an unexposed control group, with no significant differences among the intervention groups. The increased liking was maintained at 3 months follow-up (16).

### The modified intervention: Eating Veggies Is Fun!

For implementation of the program during the YMCA summer day camp, the study was renamed Eating Veggies is Fun!, because it translates well into Spanish, ¡Comer Vegetales es Divertido! Eligibility was expanded to include children aged 7 to 12 years. We aimed to enroll 50 children in a prospective cohort study with no control group. Sample size was constrained by the resources we had and by the number of children enrolled in this community venue at the time of the study. Research staff conducted the intervention and assessments in small group sessions (5 to 15 children), instead of the individual sessions used in Tiny Tastes. The baseline assessment included a 5-point emoji-like faces scale instead of Tiny Tastes’ 3-point scale with the categories Super yuck, yuck, okay, yum, super yum for 12 vegetables and 8 fruits that are commonly consumed in California and readily available in grocery stores (20). As in the original Tiny Tastes study, we assessed the children’s liking of both fruits and vegetables, but the intervention consisted of repeated tasting only of the initially disliked vegetables because the fruits were uniformly liked. Through baseline assessments with the first 17 children, we identified 5 target vegetables with the lowest average ratings: Jicama, red bell pepper, mushroom, zucchini, and sugar snap pea. Plates with small pieces of these 5 initially disliked target vegetables were offered daily for 2 weeks to all participating children in a group setting at around 9:30 AM, immediately before their regular snack time. Children were encouraged to taste the small pieces and were praised for tasting (eg, “You did a good job tasting the zucchini” or “I see that you tried a little bit of all 5 vegetables — good for you!”). Follow-up assessments were conducted immediately after the last exposures (2-week follow-up) and after 14 additional days of nonexposure to assess short-term maintenance (4-week follow-up). Research staff were trained on the study protocol and worked side-by-side with the investigators during the implementation phase. Participants received $10 for each completed assessment, up to $30 total per participant.

### Measures

**Figure 1:**
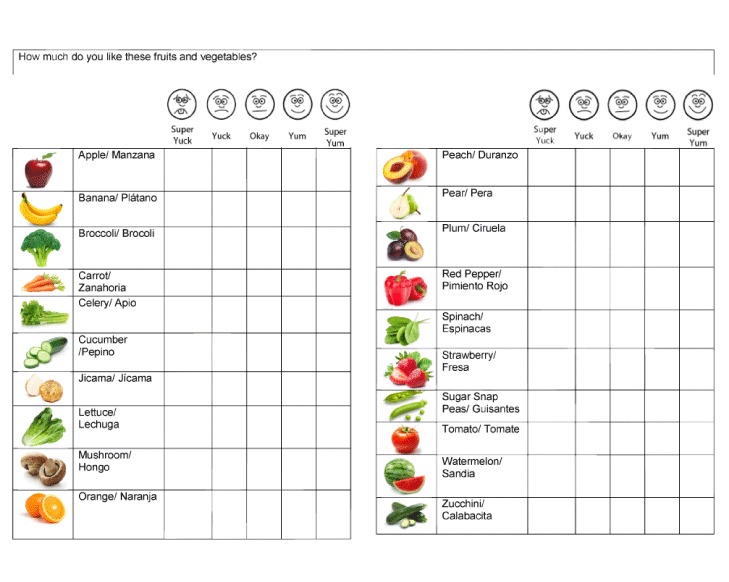
Fruits and Vegetables Assessment used in the Eating Veggies is Fun! Study, Los Angeles, California, 2015.

We assessed demographic characteristics (sex, age, race/ethnicity) at baseline. At baseline and follow-up interviews, we assessed availability of fruits and vegetables at the child’s home (never, sometimes, always), consumption of fruits and vegetables yesterday (none, 1 time, 2 times, 3 times or more) and liking of 12 vegetables and 8 fruits on a 5 point-Likert scale with faces (1 = super yuck to 5 = super yum). The fruit and vegetable assessment Likert scale was based on the research conducted by Birch and Sullivan (21) and has been used extensively in studies with children (11,22). The 2 primary outcomes were 1) liking of the 5 target vegetables and 2) how much children liked trying new foods (almost always or always; sometimes; almost never or never). All assessments were individually administered to children in English. During the fruit and vegetable assessment, children were shown pictures of the fruits and vegetables with their names in English and Spanish (Figure 1).

### Statistical analysis

We compared demographic characteristics and liking of vegetables at baseline between study completers and noncompleters using 2-sample *t* tests for continuous variables and χ^2^ tests for categorical variables. We compared liking of vegetables at baseline and follow-up using paired sample *t* tests for continuous variables, McNemar tests for dichotomous variables, and Wilcoxon signed rank tests for ordinal variables. The mean ratings for liked and initially disliked vegetables were also estimated over time using mixed effects modeling, including sex, age, and race as covariates. Analyses were conducted using IBM SPSS Statistics (version 23.0; IBM Corp) and Stata (version 14.0; StataCorp LLC).

## Results

Over 6 weeks (June 15 through July 20, 2015), we enrolled 50 children. Only 2 children from 1 family had a parent who opted them out of the study, and all children who attended the orientation agreed to participate. Although we did not record how much was consumed by each child, we did observe that all children who were enrolled in the study participated in the tasting. After repeated exposure to the 5 target vegetables, 38 children completed the 2-week follow-up assessment (76% retention). After an additional 2-week period following cessation of the vegetable tastings, 28 children completed the 4-week follow-up assessment (56% retention). More than 80% of study attrition was due to children’s absence on the day of assessment, usually because their participation in the summer camp program had ended.

Study participants were 26 boys and 24 girls, 58% were African American, 24% were Latino, and 18% reported mixed racial/ethnic backgrounds (Table 1). Their mean age was almost 9 years old. The mean baseline ratings for the 5 target vegetables ranged from 2.7 for mushrooms (between yuck and ok) to 3.5 for sugar snap peas (between okay and yum). Most children stated that they always or almost always (57%) liked to try new foods. Average baseline ratings for fruits were all positive, between 4.0 and 4.7 (yum and super yum). Children who completed the study tended to be slightly younger than noncompleters; however, completers and noncompleters did not differ in their liking for the 5 target vegetables.

**Table 1:** Baseline Information of Participants by Study Completion Status, Eating Veggies Is Fun!, Los Angeles, California, 2015

Characteristic	Total (N = 50)	Completers (n = 28)	Noncompleters (n = 22)	*P* Value^a^
**Sex, n (%)**				
Male	26 (52)	14 (50)	12 (55)	.75
Female	24 (48)	14 (50)	10 (45)	
**Race/ethnicity, n (%)**				
African American	29 (58)	20 (71)	9 (41)	.13
Latino	12 (24)	5 (18)	7 (32)	
Mixed	9 (18)	3 (11)	5 (23)	
**Age (range, 7–12 y), mean (SD)**	8.9 (1.6)	8.5 (1.4)	9.4 (1.8)	.06
**Grade next fall (range 1st–8th), mean (SD)**	4.1 (1.8)	3.7 (1.7)	4.7 (1.9)	.05
**Liking of vegetables^b^, mean (SD)**				
Jicama (n = 15 missing are excluded)	3.4 (1.7)	3.2 (1.6)	3.5 (1.8)	.61
Mushrooms	2.7 (1.5)	2.5 (1.6)	2.9 (1.5)	.32
Red bell pepper	2.9 (1.6)	3.1 (1.6)	2.7 (1.6)	.45
Sugar snap peas	3.5 (1.5)	3.4 (1.5)	3.7 (1.4)	.17
Zucchini	3.4 (1.5)	3.1 (1.6)	3.8 (1.4)	.10
**Average liking for 5 target vegetables, mean (SD)**	3.1 (1.0)	2.9 (1.0)	3.3 (0.9)	.18
**Likes to try new foods, n (%)^c^**				
Almost always or always	28 (57)	17 (61)	11 (52)	.84
Sometimes^d^	20 (41)	11 (39)	9 (43)	
Almost never or never^d^	1 (2)	0	1 (5)	

a χ^2^ tests were used for categorical variables; 2-sample *t* tests were used for continuous variables, except for average liking for vegetables, which were analyzed using mixed effects modeling with age, sex, and race as covariates.

b Rated from 1 = super yuck to 5 = super yum; 15 children who did not recognize jicama at baseline did not provide a rating; preference rating was treated as a continuous variable.

c Response was missing for 1 person.

d These 2 categories were combined for the χ^2^ test because of small cell sizes.

### Participants’ mean liking for the 5 target vegetables

**Figure 2:**
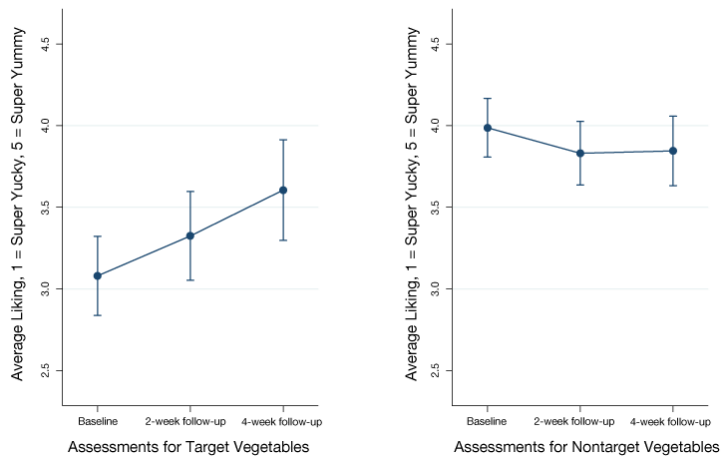
Effect of repeated exposure on mean rating for 5 initially disliked vegetables (target vegetables) and mean rating for 7 initially liked vegetables (nontargeted vegetables) (N = 50), Eating Veggies is Fun! Study, Los Angeles, California, 2015. A 5-point Likert scale was used, with 1 = super yucky to 5 = super yum.

A mixed effects modeling analysis that including sex, age, and race as covariates showed a significant increase in the liking for the 5 targeted vegetables (Figure 2). The adjusted mean liking for the 5 target vegetables was 3.1 at baseline, 3.3 at 2-week follow-up and 3.6 at 4-week follow-up (linear trend of increased liking over time was significant (χ^2^(1) = 8.66, *P* = .003). The 7 vegetables that participants already liked at baseline included broccoli, carrots, celery, cucumbers, spinach, and tomatoes. There was no significant linear trend for the adjusted mean liking for the 7 nontargeted vegetables. The means were 4.0 at baseline, 3.8 at 2-week follow-up, and 3.8 at 4-week follow-up.

**Table:** 

Assessment	Target Vegetables, Mean (95% Confidence Interval)	Nontarget Vegetables, Mean (95% Confidence Interval)
Baseline	3.1 (2.84–3.32)	4.0 (3.81–4.17)
2-week follow-up	3.3 (3.05–3.60)	3.8 (3.64–4.02)
4-week follow-up	3.6 (3.30–3.91)	3.8 (3.63–4.05)

### Changes in liking of individual vegetables and other variables

When examined singly, only the increase in liking for jicama was significant (*P* = .02). The liking for other vegetables not targeted in the study remained unchanged, except for a borderline significant drop in liking for the nontargeted vegetable celery (*P* = .05). There were no significant changes in how much children reported liking to try new foods and in the child’s reported accessibility to and consumption of fruits and vegetables “yesterday” (Table 2). In analyses that included only the 28 children who completed both the 2-week and 4-week follow-up assessment, there was no significant change in the liking of any of the vegetables examined singly and there was no change in how much children reported liking to try new foods.

**Table 2:** Baseline and 2-Week Follow-Up Comparison of Vegetable Liking and Other Variables (N = 38), Eating Veggies Is Fun!, Los Angeles, California, 2015

Characteristic	Baseline	2-Week Follow-up	Δ	*P* Value
**Liking of targeted vegetables^a^, mean (SD)**				
Jicama (N = 24)	3.2 (1.6)	4.1 (1.6)	0.92	.02^b^
Mushroom	2.5 (1.5)	2.6 (1.6)	0.14	.54^b^
Red bell pepper	3.0 (1.6)	3.3 (1.5)	0.23	.32^b^
Sugar snap pea	3.4 (1.5)	3.9 (1.5)	0.43	.22^b^
Zucchini	3.3 (1.6)	3.5 (1.7)	0.17	.66^b^
**Liking of other vegetables, mean (SD)**				
Broccoli	4.0 (1.2)	4.0 (1.2)	0.08	.63^b^
Carrot	4.5 (0.7)	4.5 (0.9)	−0.03	.84^b^
Celery	4.0 (1.1)	3.5 (1.4)	−0.46	.05^b^
Cucumber	4.0 (1.3)	3.9 (1.5)	−0.11	.57^b^
Lettuce	4.2 (1.0)	4.0 (1.2)	−0.19	.39^b^
Spinach	3.9 (1.2)	3.8 (1.4)	−0.08	.73^b^
Tomato	3.8 (1.3)	3.4 (1.5)	−0.39	.06^a^
**Likes to try new foods, n (%)**				
Almost always or always	23 (61)	16 (42)	—	.09^c^
Never to sometimes	15 (39)	22 (58)		
**Has fruits to eat at home, n (%)**				
Never	0	1 (3)	—	>.99^d^
Sometimes	13 (34)	11 (29)		
Always	25 (66)	26 (68)		
**Has vegetables to eat at home, n (%)**				
Never	0	0	—	.39^d^
Sometimes	16 (42)	12 (32)		
Always	22 (58)	25 (66)		
Don’t know	0	1 (3)		
**Ate vegetables yesterday, n (%)**				
None	6 (16)	3 (8)	—	>.99^d^
1 time	11 (29)	20 (53)		
2 times	14 (37)	10 (26)		
≥3 times	7 (18)	5 (13)		
**Ate fruits yesterday, n (%)**				
None	8 (21)	10 (26)	—	.26^d^
1 time	14 (37)	15 (40)		
2 times	12 (32)	10 (26)		
≥3 times	3 (8)	3 (8)		
Don’t know	1 (3)	0		

a Rated from 1 = super yuck to 5 = super yum. The 14 children who did not know jicama at baseline did not provide a rating. Preference rating was treated as a continuous variable.

b Paired samples *t* test.

c McNemar test.

d Wilcoxon signed rank test.

## Implications for Public Health

The modified research protocol was easy to integrate into the daily schedule of a YMCA summer day camp and the study protocol was acceptable to parents and children, as evidenced by the excellent participation rate among children who remained enrolled in the summer program. Our sample was predominantly African American, which may explain why many children were not familiar with jicama, a vegetable that is commonly eaten by Latinos. Children unfamiliar with jicama did not provide a baseline rating, thereby further reducing the sample size for this vegetable for the baseline and 2-week follow-up comparison. Despite the limited sample size, we were able to show significant increased liking for jicama among children who did provide a baseline rating, which was maintained at 4-week follow-up. Jicama was the top rated vegetable at both follow-up assessments, which suggests that repeated tasting can also increase the liking of specific unfamiliar foods. This conclusion is consistent with findings of a study by Wardle and colleagues (13).

The fact that the liking increased only for vegetables to which children were repeatedly exposed, but not for the other vegetables to which the children were not repeatedly exposed, is consistent with the hypothesis that repeated taste exposures will increase children’s liking of initially disliked vegetables. Our finding is consistent with literature showing that repeated exposure leads to increased liking for the target foods (14,16,23,24). Our finding contrasts with the finding of another study (10) that showed that verbal directives to children to eat fruits and vegetables decreased their liking for fruits and vegetables, probably because the verbal directives in that other study were applied episodically to new foods and not repeatedly enough to overcome the child’s natural food neophobia (25). Our study’s contribution is to demonstrate the feasibility of adapting an individually focused, evidence-based intervention originally involving middle-class British children for implementation in a group format and integrating it in the busy routine of a summer day camp involving children from a low-income community. Our findings also suggest that praising children for tasting the vegetables without providing a tangible award is sufficient to elicit cooperative everyday tasting of the target vegetables for 2 weeks.

Repeated exposure to the 5 vegetables did not translate into participants reporting an overall increased willingness to try other new foods. Continued broadening of the children’s repertoire of acceptable vegetables would require their sustained, repeated exposure to a wider variety of unfamiliar vegetables.

Our study was limited to a single YMCA site, which limits generalizability. Other limitations include use of a single group pretest–posttest comparison without a control group and a small sample size. The study was implemented during a summer day camp, in which children enrolled for a week at a time. Many children did not attend the YMCA camp for the whole summer, which caused substantial study attrition at the 4-week follow-up time point and prevented a longer term follow-up assessment. The program was implemented with small groups of children rather than with individual children as in the Tiny Tastes protocol, which would not be feasible in a day camp setting. We acknowledge that a strong positive or negative reaction to a vegetable from a participant could have influenced other children in the group. Our research staff tried to keep children busy during the tasting sessions to reduce peer influence on liking, similar to how children were tested in other studies (22). A popular game that children wanted to play repeatedly was to go around the table naming vegetables starting with each letter of the alphabet.

In this pilot implementation study, research staff worked with small groups of children to facilitate consistent implementation of the vegetable tasting and to administer the assessments. However, in a future implementation, camp staff could incorporate the tasting for all campers during snack time, especially if there is no need to administer assessments. Thus, vegetable tasting could be incorporated with minimal disruption to other activities and the cost of vegetables could be included in the camp food budget. We recommend that summer YMCA programs add the goal of promoting a healthy eating pattern that includes eating more vegetables into their mission statement.

This pilot study suggests that repeated vegetable tasting opportunities offered by community programs may be a practical strategy for introducing low-income, young children to new or initially disliked vegetables. The study demonstrates the feasibility of implementing a health promotion strategy that has the potential to improve population health in a community setting in an underresourced neighborhood. Given the continuing failure of traditional weight-control strategies to halt the increase in pediatric obesity (26) and given the increasing evidence that dietary patterns rich in minimally processed plant foods facilitate adult weight control (27,28), it is time to be more aggressive in broadening young children’s repertoires of acceptable fruits and vegetables.
